# Diagnosis of de novo fetal aceruloplasminemia via whole exome sequencing and fetal umbilical blood ceruloplasmin measurement

**DOI:** 10.1186/s13023-026-04445-9

**Published:** 2026-06-13

**Authors:** Pengzhen Jin, Guangmei Dai, Jiawei Hong, Shuning Han, Xiaomin Wang, Xiaohan Guo, Wensheng Hu, Yeqing Qian, Yisha Wang, Jinglan Zhang, Minyue Dong

**Affiliations:** 1 Women’s Hospital, School of Medicine, Zhejiang University, 1 Xueshi Rd, Hangzhou, Zhejiang Province 310006 China; 2 Key Laboratory of Reproductive Genetics (Zhejiang University), Ministry of Education, Hangzhou, China

**Keywords:** Aceruloplasminemia, Fetal plasma ceruloplasm measurement, Whole exome sequencing, Exon skipping, Prenatal diagnosis

## Abstract

**Background:**

Aceruloplasminemia is an autosomal recessive disorder, characterized by diabetes mellitus and progressive neurological symptoms, absent of phenotype before delivery. It is caused by mutations in *CP*, resulting in a deficiency in ceruloplasm. This study sought to diagnose sporadic fetal aceruloplasminemia via whole exome sequencing and plasma ceruloplasm measurement.

**Methods:**

Amniotic fluid and cord blood samples were obtained from a 29-year-old woman. Trio-based whole exome sequencing was performed due to right aortic arch and left subclavian artery identified in fetal ultrasound scanning, on the maternal request. Plymerase chain reaction was performed on RNA, extracted from fetal cord blood and peripheral blood of the couple. Fetal cord blood ceruloplasmin, copper, iron and transferrin were determined by enzyme linked immunosorbent assay, nephelometric method and spectrometry, respectively. Ceruloplasmin was detected as control in twelve gestational age-matched normal fetal cord blood samples.

**Results:**

No cardiac-related variants were identified, but two compound heterozygous *CP* variants (c.1306 C > T, p.(R436*) and c.3019-17T > G) were incidentally detected in the fetus. The c.1306 C > T p.(R436*) variant was a previously reported pathogenic variant that induced nonsense-mediated mRNA decay. The intronic variant (c.3019-17T > G), predicted by SpliceAI to disrupt splicing, was functionally confirmed to cause exon 18 skipping. Besides, ceruloplasmin concentrations in gestational week-matched healthy fetuses ranged from 2.75 to 56.18 mg/L (median: 14.93 mg/L, IQR: 9.96–21.43 mg/L), while it was undetectable (below the lower limit of detection) in the affected fetus. Copper and iron levels in affected fetus were within normal ranges, while transferrin levels were below the normal threshold.

**Conclusions:**

Our findings expanded the spectrum of pathogenic variants in *CP* and provided new insights into the prenatal diagnosis of the aceruloplasminemia.

**Supplementary Information:**

The online version contains supplementary material available at 10.1186/s13023-026-04445-9.

## Introduction

Aceruloplasminemia is a metabolic disease, characterized by diabetes mellitus, retinal degeneration and progressive neurological symptoms [1, 2]. The onset usually begins around age 30, and worsens at 50 [3, 4], absent of phenotype before delivery. It is mostly caused by the pathogenic variants in the *CP* gene, which is located in 3q24-q25.1, composing of 19 exons. Pathogenic variants in *CP* may result in ceruloplasmin deficiency [5, 6], leading aceruloplasminemia.

Ceruloplasmin is a plasma metalloprotein that binds more than 90% of copper, with 6–7 cupric ions in every molecule [7]. It functions as the conversion from Fe (II) transferrin to form Fe (III) transferrin [8]. The deficiency of ceruloplasmin can lead to iron accumulation in different organs, such as brain, liver and spleen, leading to systemic symptoms [2, 9]. Currently, treatment options for aceruloplasminemia are largely restricted to symptomatic management, highlighting the critical need for prenatal diagnosis in providing informed genetic counseling to expectant parents. However, fetuses affected by aceruloplasminemia typically do not present with distinctive features, which makes the identification of sporadic cases challenging in the absence of a known family history.

In the present investigation, familial trio-based whole exome sequencing (trio-WES) was conducted on the maternal request due to right aortic arch and left subclavian artery identified in fetal ultrasound scanning. No cardiac-related variants were identified, but two compound heterozygous *CP* variants (c.1306 C > T, p.(R436*) and c.3019-17T > G) were incidentally detected in the fetus. The c.1306 C > T p.(R436*) variant was a previously reported pathogenic variant that induced nonsense-mediated mRNA decay (NMD), whereas the paternally inherited c.3019-17T > G variant was initially categorized as a variant of uncertain significance (VUS). To assess the functional impact of the c.3019-17T > G variant, RNA was extracted from umbilical cord blood and subjected to reverse transcription-polymerase chain reaction (RT-PCR) and Sanger sequencing. These analyses demonstrated that the variant led to exon 18 skipping and the intronic variant was therefore upgraded as pathogenic. Furthermore, ceruloplasmin concentrations in gestational week-matched healthy fetuses ranged from 2.75 to 56.18 mg/L (median: 14.93 mg/L, IQR: 9.96–21.43 mg/L), while it was undetectable (below the lower limit of detection) in the affected fetus.

This study reports a prenatal diagnostic case of sporadic aceruloplasminemia caused by compound heterozygous variants in *CP*, expanding the spectrum of pathogenic variants in the *CP* gene. Combined functional validation and umbilical cord blood ceruloplasmin measurement provide a valuable reference for the prenatal evaluation of this rare disorder.

## Materials and methods

### Case report

**Fig. 1:**
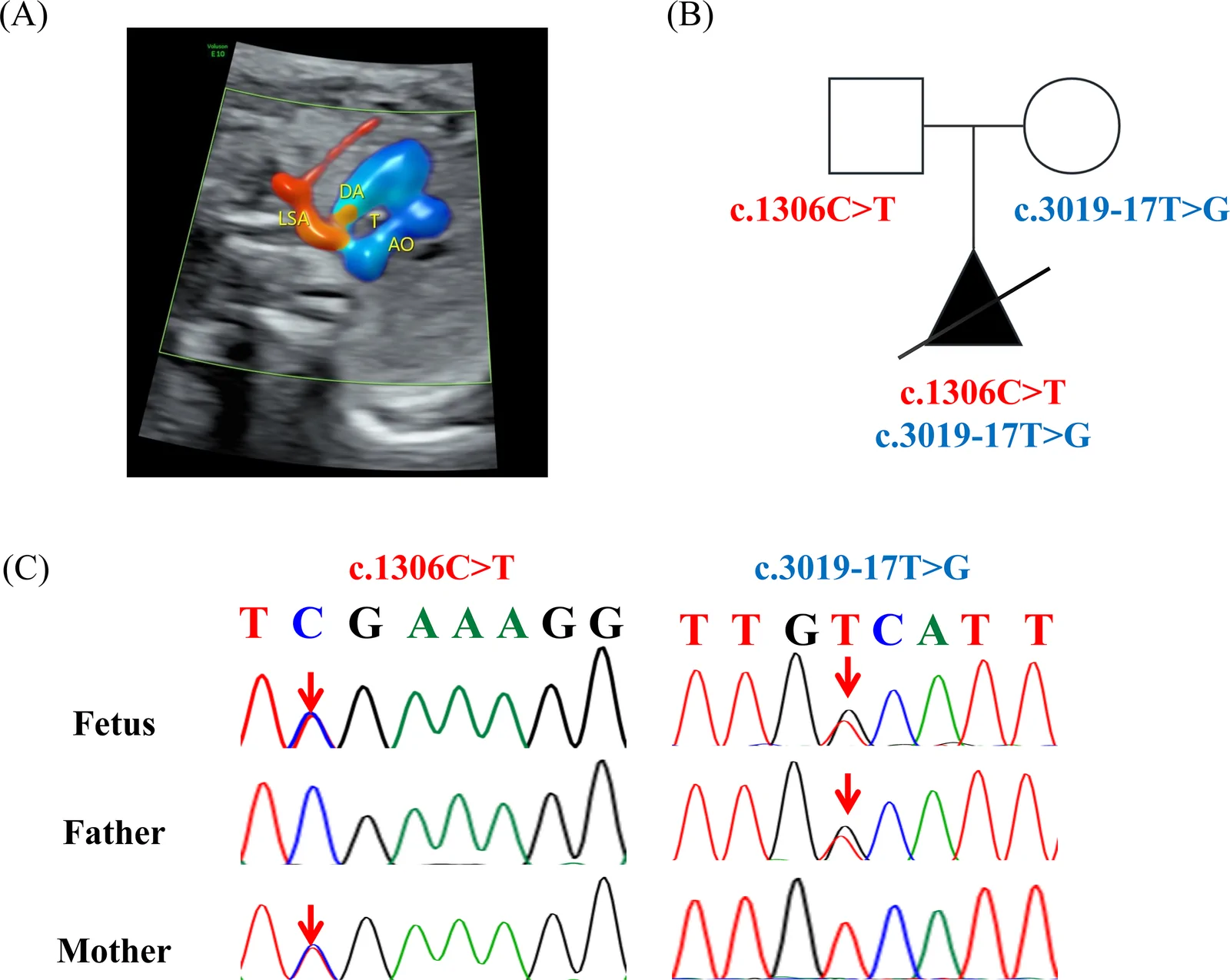
Compound heterozygous variants in *CP* identified in a non-consanguineous family. (**A**) Right aortic arch with aberrant left subclavian artery in the fetus. (**B**) Family pedigree. (**C**) Sanger sequencing validation of *CP* variants

The proband was the first conceptus of a non-consanguineous Chinese couple. Noninvasive prenatal testing indicated low risks for trisomy 21, 18 and 13. Routine prenatal ultrasound scans revealed a right aortic arch and aberrant left subclavian artery at 20 weeks of gestation (Fig. 1A). It is a vascular ring malformation with no discernible adverse impacts on fetal cardiac function or systemic circulation. All echocardiographic parameters were within gestational age reference ranges, and no hemodynamic disturbance was noted. The tracheoesophageal compression is rare. Symptomatic cases are amenable to surgical vascular ring release, whereas asymptomatic cases only require regular pediatric cardiology follow-up.

Trio whole-exome sequencing (trio-WES) is not a routine clinical indication for this isolated malformation, given insufficient evidence for an underlying genetic etiology; however, transabdominal amniocentesis was performed under real-time sonographic guidance at 26 weeks, at the request of the pregnant woman. Fetal karyotyping and chromosome microarray showed normal results (data not shown). Two compound heterozygous variants in *CP* were incidentally identified through trio-WES.

The study was approved by the Ethics Committee of Women’s Hospital, School of Medicine Zhejiang University (IRB-20240266-R). The couples provided written informed consents.

### Amniocentesis and fetal karyotyping

Amniocentesis was carried out at 26 weeks of gestation under real-time sonographic guidance. A total of 30 ml amniotic fluid was collected and cells were cultured with BIOAMF-2 Complete Medium (Biological Industries, Cromwell, CT). Chromosome karyotyping was conducted on metaphase preparations using the GSL-120 CytoVision platform (Leica, German). The DNA samples collected from the amniotic fluid of the fetus and the peripheral blood of the couple were frozen at -80℃ until trio-WES analysis.

### Cordocentesis

Transabdominal umbilical cord blood sampling was performed on the affected fetus under real-time ultrasound guidance. A total of 4 mL of cord blood was collected. Of this, 1 mL was used for total RNA and DNA extraction. An additional 2 mL was centrifuged at 4 ℃ at 3000 rpm for 10 min to separate the plasma. The remaining blood was collected in heparin anticoagulation vacuum tube.

12 eligible controls were enrolled from pregnancies that underwent for further prenatal diagnosis due to advanced maternal age or abnormal non-invasive prenatal screening (NIPS) findings. Subsequent comprehensive assessments confirmed the absence of structural malformations, genetic abnormalities and other medical anomalies. The control group was strictly gestational age-matched (28–32 weeks) to the affected fetus. Gestational ages were assessed by the last menstrual period. All samples were obtained with parental informed consents.

### Whole exome sequencing

DNA samples from the fetus and the couple were sequenced using the MGISEQ-2000 platform (MGI Tech) following the standard workflow. The KAPA HyperExome Exome Enrichment Kit (Roche Diagnostics) was used for exome capture in this study. Custom capture probes from this kit were hybridized with 9 barcoded DNA libraries at 65 ℃ for 12 h, followed by probe washing, elution, and post-PCR amplification of captured products, which constituted the core exome enrichment step of the sequencing workflow. Clean reads were aligned to the hg19 reference genome using BWA, and variants including SNVs and Indels were identified using GATK. Variant filtering and annotation were performed against multiple databases, including NCBI dbSNP 147, dbNSFP v2.9.1, ESP6500, HapMap, the 1000 Genomes Project, and a database of 10 Chinese healthy adults, using the BGI-Varanno algorithm. Copy number variations (CNVs) were analyzed using exomeDepth (for exonic CNVs) and CNVkit (for large CNVs > 1 Mb), and dynamic mutations were detected using ExpansionHunter. Pathogenicity of the identified variants was assessed according to the guidelines of the American College of Medical Genetics and Genomics (ACMG), combined with disease databases including ClinVar, OMIM, and HGMD. Only variants relevant to the patient’s clinical manifestations were reported.

### PCR amplification, agarose gel electrophoresis and Sanger sequencing

Genomic DNA was isolated from umbilical and peripheral blood using the QIAamp DNA Blood Mini Kit (Qiagen, Hilden, Germany) following the manufacturer’s protocol. Total RNA was extracted using TRIzol reagent (Takara, Japan). Nucleic acid concentrations were quantified using the NanoDrop 2000. Complementary DNA (cDNA) was synthesized from total RNA with a commercial reverse transcription kit (Takara, Japan) according to the manufacturer’s instructions.

For genomic DNA amplification, locus-specific primers were designed for the variant regions: *CP*: c.1306 C > T (DNA), 5′-TTTTCCCCCTGCAGTGACTC-3′ (forward) and 5′-AGTGCAGAGGCTCACAACTT-3′ (reverse); *CP*: c.3019-17T > G (DNA), 5′-GGCCACAGCTGAGTTCCTTT-3′ (forward) and 5′-TTTGGGGTTGTCTTGGGCTT-3′ (reverse). For cDNA amplification targeting the corresponding variants, primers were 5′-TCCCAGGACTCTCCATGTGT-3′ (forward) and 5′-CTCACCCCAATCGGCTCAAT-3′ (reverse) for the nonsense variant (c.1306 C > T) and 5′-TGGCCCCCTG ATTGTTTGTC-3′ (forward) and 5′-CCTTCCCTCATACCTTGCCC-3′ (reverse) for the intronic variant (c.3019-17T > G).

PCR was performed with an initial denaturation at 94 °C for 5 min, followed by 40 cycles of 94 °C for 30 s, 60 °C for 30 s, and 72 °C for 60 s, with a final extension at 72 °C for 10 min. PCR products were separated by 1.5% agarose gel electrophoresis at 100 V for 25 min on a Biometra electrophoresis system (Germany). Amplified fragments were subjected to Sanger sequencing on an ABI 3130 Genetic Analyzer.

### Protein structure homology modeling

The three-dimensional structure of the target protein was predicted using the SWISS-MODEL web server (https://swissmodel.expasy.org/). The amino acid sequence of the protein was submitted as the input query. The server automatically searched the SWISS-MODEL Template Library (SMTL) to identify structurally resolved homologous templates via integrated BLAST and HHblits algorithms. Candidate templates were ranked by the Global Model Quality Estimate (GMQE) and Quaternary Structure Quality Estimate (QSQE), and the top-rated template with high sequence identity and optimal structural reliability was selected for model construction. Atomic-level three-dimensional models were generated based on the optimized target-template sequence alignment. The quality and stereochemical validity of the final model were further evaluated using the QMEAN scoring function to ensure computational reliability and structural rationality.

### Biochemistry assay

Plasma ceruloplasmin concentrations were determined by enzyme linked immunosorbent assay (Huamei, Wuhan, China) and transferrin was measured using immunoturbidimetric assay in cord blood. Copper and iron was determined by the inductively coupled plasma mass spectrometry (Agilent Technologies, China) in whole blood. All these procedures were conducted according to the manufacturers’ instructions.

## RESULTS

### Identification of compound heterozygous variants in *CP*

**Fig. 2:**
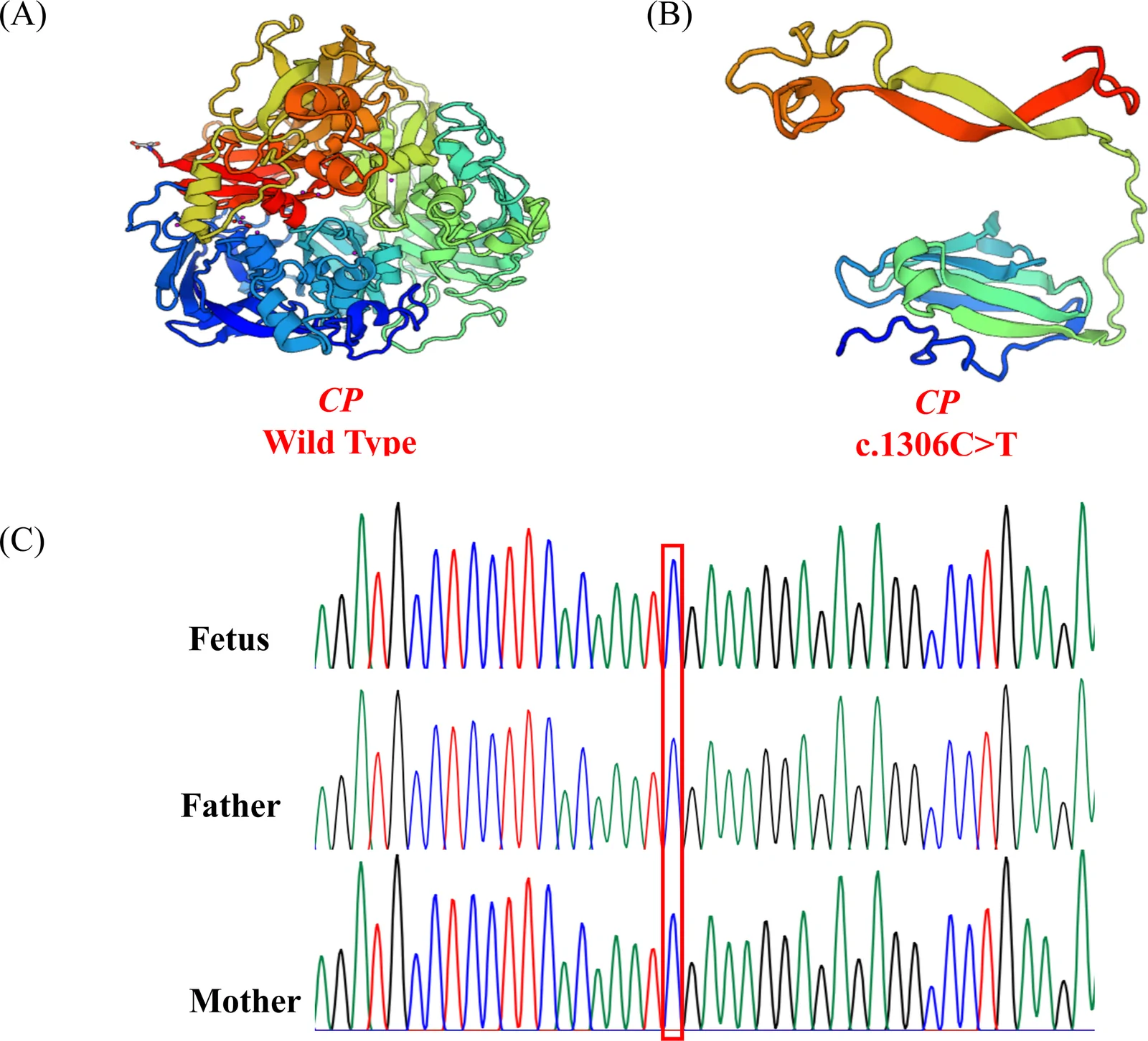
Pathogenic characterization of the *CP*:c.1306 C > T variant. (**A**) Predicted protein structure of wild-type CP. (**B**) Predicted protein structure of the *CP*:c.1306 C > T variant. (**C**) cDNA sequencing results in family members

Two compound heterozygous variants of *CP* (NM_000096.4) were identified in the fetus through trio-WES (Fig. 1B-C). The maternally inherited c.1306 C > T p.(R436*) variant introduces a premature stop codon, resulting in early termination of translation (Fig. 2A-B). Frequencies of this variant, 0.0000164 and 0.0000326 were reported in the ExAC and gnomAD-exome databases, respectively, with no occurrence recorded in the 1000 Genomes Project. It was reported in the literature as disease-causing and shown to introduce NMD [10]. Sanger sequencing of locus-specific cDNA amplicons detected only the wild-type sequence in all three samples but not the mutant allele, substantiating the presence of NMD (Fig. 2C). According to ACMG guidelines, this variant is classified as pathogenic.

The c.3019-17T > G variant, which was paternally inherited, has not been reported in the 1000 Genomes Project, ExAC, or gnomAD-exome databases. Predictive tools, including SpliceAI, Spidex, and MaxEntScan, indicated that this variant may affect splicing. Based on ACMG guidelines, this variant is classified as VUS.

### Confirmation of the exon 18 skipping

**Fig. 3:**
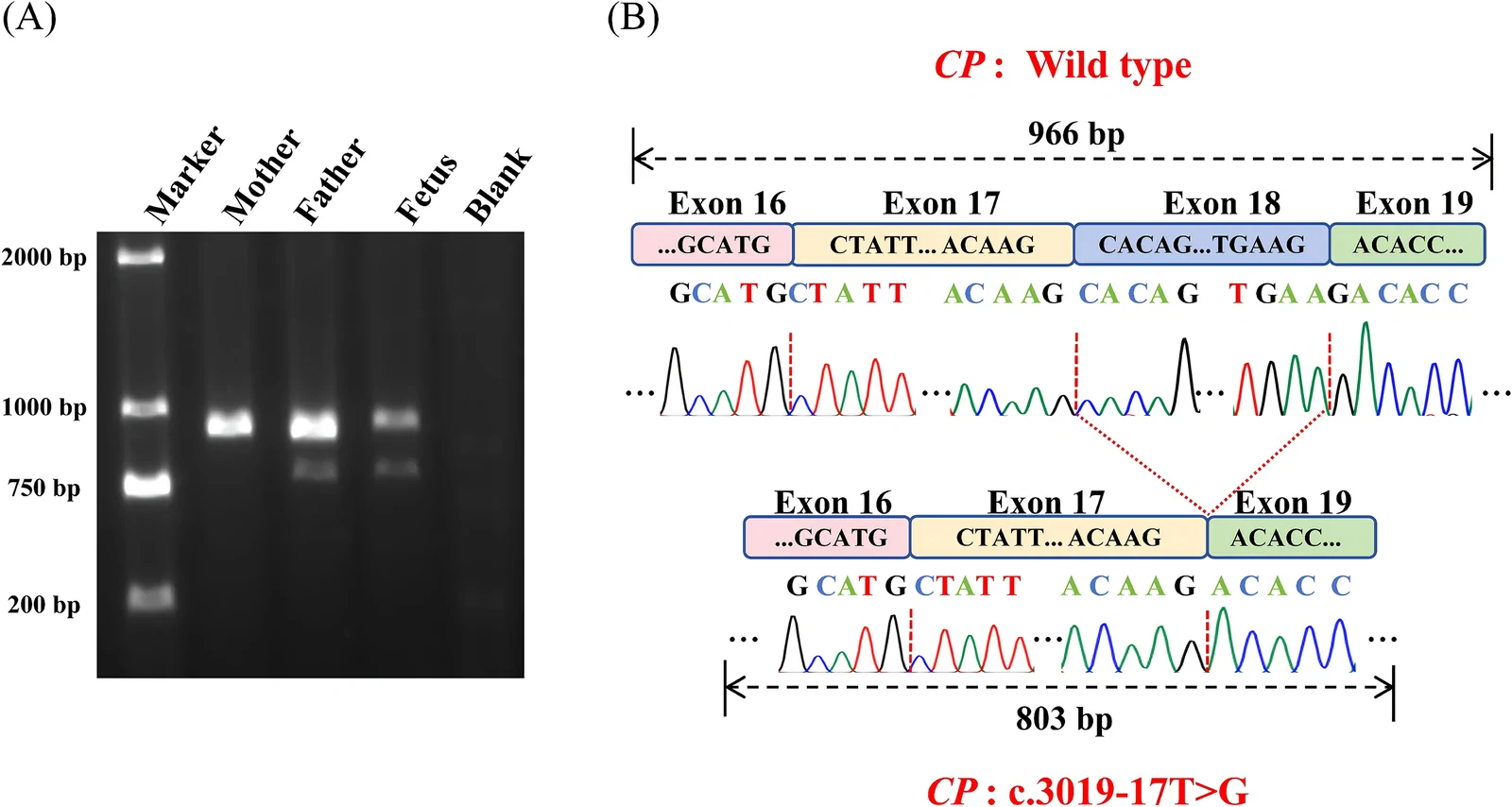
Pathogenic characterization of the *CP*:c.3019-17T > G variant. (**A**) Gel image of RNA splicing assay. (**B**) Sanger sequencing results

Based on the gel electrophoresis results (Fig. 3A), the bands corresponding to the fetus and father were 966 bp and 803 bp in size, respectively. The mother’s sample displayed a single band, approximately 966 bp in size. Subsequent Sanger sequencing and BLAST analysis in NCBI revealed that the 966 bp band contained sequences from part of exon 16, exon 17, exon 18, and part of exon 19, while the 803 bp band included sequences from part of exon 16, exon 17, and part of exon 19. Notably, when compared to the mother’s sequence, the bands corresponding to the proband and the father exhibited complete skipping of exon 18 (Fig. 3B).

### Deficiency of ceruloplasmin in umbilical blood

As is shown in Table 1, ceruloplasmin concentrations in normal fetal samples were ranged from 2.75 mg/L to 56.18 mg/L (median: 14.93 mg/L, IQR: 9.96–21.43 mg/L), while undetectable in affected fetal sample (the detection limitation 0.156 mg/L). In addition, the concentration of copper, iron and transferrin were 18.59 umol/l, 6.84mmol/l and 0.84 g/l, respectively.

**Table 1:** The concentrations of the ceruloplasmin

Number	Gestational week	Ceruloplasmin concentrations(mg/L)
1	29	2.75
2	29	7.43
3	29	9.35
4	32	10.56
5	28	12.65
6	28	13.64
7	31	16.21
8	31	16.64
9	32	21.4
10	31	21.46
11	28	25.12
12	32	56.18
adult women	/	300
adult men	/	438
**affected fetus**	**32**	**undetected**

## Discussion

In this study, we identified two compound heterozygous variants in *CP* in a fetus, a truncating pathogenic variant and an intronic variant initially classified as VUS by prenatal trio-WES. Subsequent RT-PCR analysis of fetal RNA followed by Sanger sequencing demonstrated that the intronic variant caused exon 18 skipping, leading to its reclassification as pathogenic. Fetal umbilical cord blood ceruloplasmin concentration was below the lower limit of detection, markedly lower than the reference range of gestational week-matched healthy fetuses, which provided biochemical evidence supporting the prenatal diagnosis of aceruloplasminemia. We report a rare prenatal diagnosis of sporadic aceruloplasminemia in a fetus without a relevant family history. Our findings further expand the pathogenic mutational spectrum of *CP* and support that the combination of functional validation and fetal ceruloplasmin measurement may serve as a useful reference for prenatal evaluation and genetic counseling in similar cases.

Inborn errors of metabolism are genetic disorders characterized by enzyme deficiencies that disrupt various metabolic pathways, leading to the accumulation of the upstream substrates and the insufficiency of the products [11, 12]. Thus, biochemical analyses are crucial for identifying the underlying metabolic etiology. Fetal manifestations are highly variable, depending on the different metabolic pathways active in fetal period [13]. Most of the disease are absent of prenatal phenotypes, making the prenatal identification a great challenge [13].

Amniotic fluid biochemical assays have proven to be effective for the prenatal diagnosis of metabolic disorders [14]. For instance, elevated concentrations of propionylcarnitine, acetylcarnitine, methylmalonic acid, and methylcitrate in amniotic fluid are consistent with the diagnosis of methylmalonic acidemia [15]; reduced enzyme activity in the amniotic fluid supernatant has been demonstrated as a marker for the prenatal diagnosis of mucolipidosis [16]; free sialic acid in amniotic fluid supernatant can be used for screening of sialic acid storage diseases [17]. However, prenatal biochemical testing for the diagnosis of aceruloplasminemia has not been previously reported.

Aceruloplasminemia is a metabolic disease, characterized by diabetes mellitus, retinal degeneration and progressive neurological symptoms. The onset usually begins around age 30, and worsens at 50, absent of phenotype before delivery. It is mostly caused by the variants in the *CP* gene. By disrupting *Cp*, a deficiency of ceruloplasmin and progressive accumulation of iron in the liver and spleen of mice were observed at one year of age [18]. The movement of iron was impaired in mice reticuloendothelial and hepatocyte cells, making substantial iron storing in it. Variants identified in patients were previously reported in exon 3, 7, 18 and intron 1, 3, 6, 18 [3, 4, 19–22], mostly leading to the truncating or skipping (Supplementary Material Table 1. Characteristics of patients with *CP* variants). The functional disruption in *CP* led to ceruloplasm deficiency and iron accumulation, which was highly related to the symptom of aceruloplasminemia.

Aceruloplasminemia is phenotypically heterogeneous and ceruloplasm measurement is critical for the disease diagnosis. Ceruloplasmin cannot cross the maternal-fetal barrier via the placenta [23]. All ceruloplasmin is synthesized endogenously and cannot be compensated by maternal or placental metabolism. It can be detectable in the yolk sac and increases throughout the gestation [24]. Previous studies have quantified ceruloplasmin levels in cord blood of term and preterm neonates, establishing preliminary reference values for postnatal ceruloplasmin status in early life [25]. Notwithstanding these contributions, there remains a critical gap in the literature regarding ceruloplasmin levels during the fetal period—specifically in the first and second trimesters of human pregnancy. To date, no published studies have systematically determined ceruloplasmin concentrations in fetal serum across these early gestational stages. This absence of fetal ceruloplasmin data precludes the establishment of normative reference ranges for fetal ceruloplasmin and severely limits our ability to evaluate whether fetal ceruloplasmin levels fall within physiologically normal ranges throughout gestation.

As was shown in our experiments, normal adult ceruloplasmin concentrations ranged from 300 to 400 mg/L, aligning with prior pulications [10]. In contrast, most control fetal ceruloplasmin levels cluster at 9.96–21.43 mg/L, reflecting physiological heterogeneity in fetal hepatic ceruloplasmin synthesis. The exceptionally low value (2.75 mg/L) and the markedly elevated value (56.18 mg/L) most probably stem from individual variation or experimental operational factors. Notably, ceruloplasmin levels in affected fetuses were significantly lower than those in gestational age-matched normal controls. This striking reduction highlights the potential utility of cordocentesis-based ceruloplasmin measurement as an adjunctive diagnostic tool for ceruloplasmin deficiency disorders during the fetal period. However, critical preanalytical and methodological considerations must be addressed to ensure the reliability of this approach. Strict adherence to cordocentesis technique, including careful avoidance of amniotic fluid contamination and meticulous sample handling to prevent hemolysis, is therefore essential to minimize these artifacts and preserve diagnostic validity.

Finally, while our findings provide the first empirical framework for fetal ceruloplasmin reference ranges, further studies with expanded sample sizes across a broader spectrum of gestational ages are warranted to refine these preliminary values. Larger cohorts will enable more precise stratification of ceruloplasmin levels by gestational week, reducing the impact of physiological heterogeneity and enhancing the clinical utility of this biomarker for prenatal evaluation of aceruloplasminemia.

## Supplementary Information

Below is the link to the electronic supplementary material.

Supplementary Material 1

Supplementary Material 2

## Data Availability

The data that support the findings of this study are openly available in the BIG Submission Portal repository, reference number PRJCA030003.
